# Dynamic models of stress-smoking responses based on high-frequency sensor data

**DOI:** 10.1038/s41746-021-00532-2

**Published:** 2021-11-23

**Authors:** Sahar Hojjatinia, Elyse R. Daly, Timothy Hnat, Syed Monowar Hossain, Santosh Kumar, Constantino M. Lagoa, Inbal Nahum-Shani, Shahin Alan Samiei, Bonnie Spring, David E. Conroy

**Affiliations:** 1 School of Electrical Engineering and Computer Science, The Pennsylvania State University, University Park, PA 16802 USA; 2 Department of Preventive Medicine, Northwestern University Feinberg School of Medicine, Chicago, IL 60611 USA; 3 Department of Computer Science, University of Memphis, Memphis, TN 38152 USA; 4 Institute for Social Research, University of Michigan, Ann Arbor, MI 48106 USA; 5 Department of Kinesiology, The Pennsylvania State University, University Park, PA 16802 USA

**Keywords:** Predictive markers, Lifestyle modification, Risk factors

## Abstract

Self-reports indicate that stress increases the risk for smoking; however, intensive data from sensors can provide a more nuanced understanding of stress in the moments leading up to and following smoking events. Identifying personalized dynamical models of stress-smoking responses can improve characterizations of smoking responses following stress, but techniques used to identify these models require intensive longitudinal data. This study leveraged advances in wearable sensing technology and digital markers of stress and smoking to identify person-specific models of stress and smoking system dynamics by considering stress immediately before, during, and after smoking events. Adult smokers (*n* = 45) wore the AutoSense chestband (respiration-inductive plethysmograph, electrocardiogram, accelerometer) with MotionSense (accelerometers, gyroscopes) on each wrist for three days prior to a quit attempt. The odds of minute-level smoking events were regressed on minute-level stress probabilities to identify person-specific dynamic models of smoking responses to stress. Simulated pulse responses to a continuous stress episode revealed a consistent pattern of increased odds of smoking either shortly after the beginning of the simulated stress episode or with a delay, for all participants. This pattern is followed by a dramatic reduction in the probability of smoking thereafter, for about half of the participants (49%). Sensor-detected stress probabilities indicate a vulnerability for smoking that may be used as a tailoring variable for just-in-time interventions to support quit attempts.

Cigarette smoking is the leading preventable cause of death in the United States, responsible for about one in five deaths annually^1^. The costs of direct medical care for adult smokers, lost economic productivity caused by smoking-related disability and premature mortality, and secondhand smoking exposure exceed $300 billion per year in the United States^2,3^. Comprehensive smoking control programs require a clear understanding of the dynamics of smoking triggers and behaviors; however, few studies have attempted to describe those systems. This study used high-frequency sensors and novel markers of both stress and smoking to describe system dynamics and then simulate how stress influences subsequent risk for smoking.

Stress and negative emotions have long been thought to be motives for smoking^4–7^. Stress represents the nonspecific response of the body to a demand^8^. It manifests as distress and negative affect when the perceived demand exceeds the individual’s coping potential^9^. Smokers identify stress as a reason for smoking^4–6^. One reason may be that they learn that nicotine self-administration can dispel negative affective states that are caused by withdrawal and produce acute psychological benefits such as improved mood and concentration^10–12^.

Evidence based on a variety of methods supports the concept that stress-induced negative affect is positively associated with smoking^13–18^. For example, the acute stress of a terrorist attack in New York City increased smoking rates among residents^19^. On a faster time scale, Shiffman and colleagues found a positive association between repeated assessments of daily stress and smoking^14^. Affect dysregulation, particularly negative mood variation, has also emerged as a risk factor for future smoking escalation^15^. These findings point to stress responses as a target for behavioral interventions to reduce smoking and as a potential tailoring variable for interventions to reduce stress-related smoking.

The stress and smoking literature have three gaps that need to be addressed to inform intervention development efforts. First, most of the literature assessing links between stress or negative affect, on the one hand, and smoking, on the other hand, involves data collected during a quit attempt^20–24^. Assessments of stress taken after quitting smoking reflect both physiological arousal due to daily life experiences and distress due to nicotine withdrawal. Stress-smoking dynamics may be able to be characterized more consistently prior to a quit attempt before prolonged nicotine withdrawal disrupts baseline stress-smoking dynamics. These dynamics can expose the duration of vulnerability between the onset of a stress response and smoking behavior. That information can be used to tailor decision rules (i.e., general or person-specific algorithms) for just-in-time-adaptive smoking cessation interventions in the future.

Second, available analyses have relied on periodic measurement of stress and smoking based on self-reports. However, these self-report assessments are made too infrequently to capture sufficient granularity in continuous stress dynamics leading to and following smoking events. In addition, self-reports are vulnerable to bias from memory errors, psychological interference, and self-presentational concerns^13,25^. The present study uses sensor-based markers of both stress and smoking to obtain more intensive data that can reveal the dynamics of stress and smoking.

Third, no attempts have been made to study the dynamics of stress in the moments immediately prior to and following smoking events. To date, stress has not been measured continuously and data have typically been limited to a single data point about stress prior to a smoking event. The availability of sensors and digital markers of stress creates new possibilities for monitoring stress continuously so that a time series of stress measurements can be used to predict smoking events and to characterize changes in stress after smoking. In this study, intensive longitudinal digital assessments afforded sufficiently continuous, granular portrayal of stress dynamics to develop a model informed by stress immediately before, during, and after smoking events. This approach can provide the foundation for predicting the temporal response of individuals to stress as a basis for developing evidence-based, just-in-time smoking cessation interventions.

Recent advances in wearable sensors and mHealth markers enable continuous monitoring and detection of stress states and smoking behavior. Useful measurements may include electrical activity of the heart, breathing dynamics, hand movements, and physical activity. Continuously-measured stress states (i.e., inferred from physiological arousal in the absence of physical activity) and smoking behavior sampled at high frequencies (e.g., minute-level) via wearable devices require different tools for their analysis than more sparsely distributed periodic data from ecological momentary assessments to characterize system dynamics. Understanding these dynamics will provide a foundation for adaptive treatments on the individual level so that interventions can be delivered at the moments of greatest need. High-frequency data indicators of an outcome (e.g., minute-level smoking behavior) are not only dependent on the present moment of the candidate predictor (e.g., stress) but are also correlated with past values of that predictor. In other words, a stress episode can affect both the probability of smoking immediately and increase the smoking likelihood in the minutes following the stress episode. Therefore, data signifying present moments of smoking need to be connected to data from immediately prior values of stress to model the stress-smoking responses. Dynamical systems provide the necessary tools to model this so-called “memory effect”, that is, the effect of past and present values of the predictor on the future outcome. In this context, the system is a process that connects past and present values of a predictor (stress) to an outcome (smoking).

Smoking is a complex, dynamic behavior. It can be influenced by a variety of factors over time and is not necessarily the instantaneous result of stress (or other causes). Given the potential for a delay (a time shift or lag) of unknown duration between stress and smoking, existing analytic tools for behavioral studies mentioned above are not well suited to using all of the information in high-frequency intensive longitudinal data and providing a comprehensive description of the relation between system variables^26,27^. This problem is acute when the length of the delay is unknown and may vary for different systems or people.

Control systems engineering tools have been applied to regulate complex, dynamic systems (e.g., air conditioning in homes, cruise control in cars, flight in aircraft). In general, a system is a process that produces repeated outcomes in response to repeated predictors. Identifying a model of the relations between variables (i.e., predictors and outcomes) that unfold over time (hereafter, a system) is the starting point for developing algorithms to make informed decisions for controlling the system. System identification techniques are a set of tools that can be used to characterize the relationship between predictors and outcomes over time. Often the identified models need to be tuned for the specific system (e.g., for a specific home, vehicle or aircraft) and this tuning is particularly important for systems describing human behavior, which have a high level of uncertainty due to multiple causes. We propose to leverage individual intensive longitudinal data to identify person-specific models that describe dynamic relations between stress and smoking for each individual. Models obtained by these techniques provide the parameters needed to potentially develop person-specific algorithms (i.e., decision rules) for adapting the delivery of a just-in-time intervention based on person-specific vulnerabilities. Such models could realize the potential for personalized behavior medicine to provide people with the right treatment only at the moment when they need it, based on the person-specific dynamics. This approach allows for greater flexibility in designing just-in-time interventions because it can provide evidence-based, tailored—even person-specific—decision rules when stress-smoking systems differ between people (e.g., due to varying model complexity or input salience). In this paper, we focus on stress as a potential tailoring variable for just-in-time interventions to prevent smoking behavior and model stress-smoking systems. Previously, control systems engineering tools were used to model and design interventions for gestational weight gain and physical activity^28–32^, but we apply them here to characterize the dynamics of the stress-smoking responses.

System identification methods—techniques to fit a dynamic model from longitudinal measurements of a predictor to an outcome—have been used to evaluate smoking cessation treatments^33^. These methods have been combined with model predictive controllers—controllers (decision rules) that rely on dynamic models of a process obtained by system identification to determine the “right” inputs to the process—to provide a foundation for designing adaptive intensive smoking interventions^34,35^. Simulations by Lagoa et al. revealed that treatment outcomes could be improved by delivering real-time interventions when and where needed^35^. Bekiroglu et al. also represented the relations between tobacco withdrawal-related processes over time using dynamical models^36^. These works demonstrated the feasibility of applying system identification tools to model problems involving smoking. Moreover, they provided preliminary results on how the identified models can be used to develop just-in-time interventions. This study will provide the first application of system identification tools to model person-specific stress-smoking systems and will provide the basis for applying model predictive control tools to design an adaptive intensive, smoking intervention tailored on stress.

The prior smoking-related applications involved continuous outcome measures, leading to relatively straightforward modeling procedures. However, in our system, the predictor (stress) is represented by a continuous score and the outcome (smoking event) is represented by a binary value. Therefore, another contribution of this work is the development of a modeling technique to describe relations between our continuous predictor and a binary outcome in an efficient way. Model identification of a connection of a linear system with a hard nonlinearity is known to be a complex problem because the resulting optimization problem is nonconvex and, hence, hard to solve numerically. In this paper, we provide computationally efficient convex relaxations aimed at obtaining the lowest complexity model compatible with the data available. Furthermore, to the best of our knowledge, system identification methods have never been applied to describe the person-specific dynamics of stress-smoking responses with a binary outcome.

Data for the current research were drawn from the parent Sense2Stop clinical trial, a micro-randomized trial of 75 smokers that used wearable sensors to optimize a just-in-time-adaptive stress-management intervention for smoking relapse prevention^37^. The Sense2Stop study, described elsewhere^37^, used wearable devices to collect intensive longitudinal data and applied algorithms to estimate the probability of stress (cStress) at every minute and detect smoking events (puffMarker)^38,39^. These two algorithms have been used to evaluate stress and smoking lapses in another study using independent data conducted by some of the current paper’s authors^40^. We applied system identification techniques from control systems engineering to model person-specific dynamic relations between stress and smoking. In other words, we modeled individual behavior using only data recorded from that person, and, therefore, determined a different system/model for each participant. Analyzing stress-smoking relations, especially at the moments preceding and following a smoking event and generating person-specific model parameters in the present study, provides a future basis for designing person-specific, tailored smoking cessation treatments from the parameters linking vulnerable moments (when stress is likely to lead to smoking) to intervention decisions.

## Results

### Sample characteristics

Characteristics of 45 participants with sufficient data to model person-specific dynamic relations between stress and smoking in the present study are summarized in Table 1 (data processing steps that led to the final analytic sample are described in the “Methods” section). A summary of their preprocessed and analyzed data can be found in Supplementary Tables 1 and 2. These quantities include the numbers of samples in the original dataset, samples after interpolation (with missing samples ≤2), chunks (continuous series of samples) based on the interpolated dataset, chunks after removing the small chunks (size <5 samples), and samples after excluding the small chunks. Prior to interpolation, the average total duration of data from each participant sampled in the dataset was 723.1 min (SD = 323.6; range = 79–1452) over 3 days; after interpolation, the average total duration of data from each participant increased to 887.9 min (SD = 385.5; range = 90–1776) over 3 days (wear time for many participants was <11.2 h/day). The interpolated samples were divided into chunks with a mean number of 76.2 chunks/participant (SD = 33.1; range = 3–150). After removing data chunks <5 min long, the mean number of data chunks/participants used for modeling was 40.5 (SD = 16.45; range = 3–80). The mean total duration of samples used for system identification was 819.9 min (SD = 375.8; range = 90–1716).

**Table 1:** Characteristics of the participants.

Demographic characteristic	*n* (%)	M (SD)	Observed range
Age		42.6 (14)	20–64
Sex			
Male	23 (51%)		
Female	22 (49%)		
Race			
American Indian/Alaska Native	0 (0%)		
Asian	1 (2%)		
Native Hawaiian/Other Pacific Islander	0 (0%)		
Black/African American	23 (51%)		
White	14 (31%)		
Two or more races	5 (11%)		
Other	2 (4%)		
Ethnicity			
Not Hispanic/Latino	40 (89%)		
Hispanic/Latino	5 (11%)		
Number of cigarettes per day			
10 or less	28 (62%)		
11–20	12 (27%)		
21–30	3 (7%)		
31 or more	2 (4%)		
Age of starting to smoke?		18.3 (6.5)	7–45

### Person-specific dynamical modeling

**Fig. 1:**
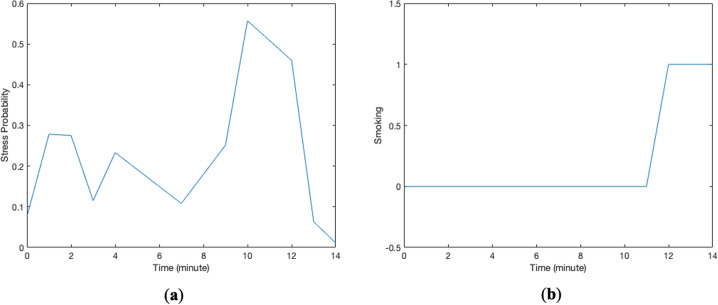
Data chunks are uninterrupted time series for variables in the system. These two plots represent changes in **a** stress probabilities and **b** a binary smoking outcome for one data chunk.

**Fig. 2:**
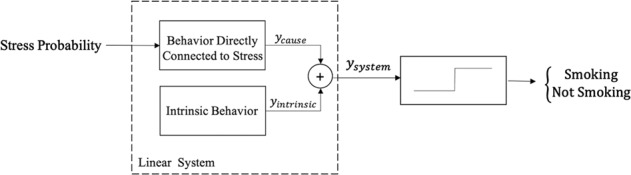
System configuration for dynamical model that integrates a linear system and a nonlinearity.

**Fig. 3:**
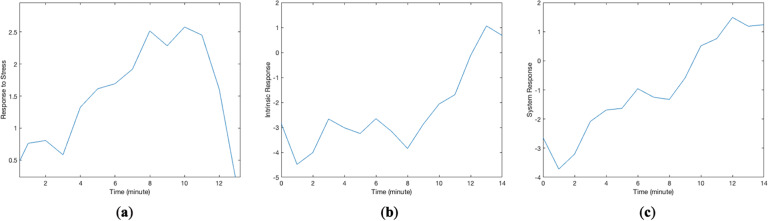
Different responses of the identified system for one data chunk. **a** Response of the system to predictor (stress), y_cause_, **b** intrinsic response of the system, y_intrinsic_, and **c** system response, which is the sum of response to predictor and intrinsic response, y_system_.

The modeling strategy used in this paper was person-specific dynamical system modeling. We use the term “system” to represent a longitudinal process that produces repeated outcomes in response to repeated predictors. Stress probability was considered as the predictor and smoking as the outcome. Figure 1 shows hypothetical changes in the candidate predictor (stress probability) and outcome (smoking events) for one data chunk consisting of 15 samples recorded on a minute scale. Stress probabilities ranged from 0 to 1; smoking values of 0 and 1 indicate non-smoking and smoking events at that minute. The modeling objective is to identify a system that represents the relation between these two longitudinal signals. As shown in the system in Fig. 2, the first part of the model is a linear system with stress probability as the predictor and “response of the system,” denoted by $y_{system}$, as the linear system response^41^. The linear system response, $y_{\rm{system}}$, is the summation of the behavior of the system which is directly connected to stress, $y_{\rm{cause}}$, and the intrinsic behavior of the system, $y_{\rm{intrinsic}}$. In the context of dynamical model of the stress-smoking responses, intrinsic response, $y_{\rm{intrinsic}}$, can be thought of as the estimate of smoking odds when the participant is not experiencing stress. Figure 3 represents the predictor response, intrinsic response, and the overall response of the system for the data chunk in Fig. 1. *Predictor response* is a behavior of the system which is directly connected to stress ($y_{\rm{cause}}$), *intrinsic response* represents the odds of smoking when stress is zero ($y_{\rm{intrinsic}}$), and the *system response* plot represents the summation of the response of the system to stress and intrinsic response ($y_{\rm{system}}$). Whenever the value of $y_{\rm{system}}(t)$ equals or exceeds the threshold of 1, there is likely to be a smoking minute in the outcome, and if $y_{\rm{system}}(t) < 1$, there is no smoking occurrence. Note that, in the proposed approach, we aimed at modeling individual behavior and, therefore, a different system or model is identified for each participant. The right-hand side block in the system configuration depicted in Fig. 2, which is called “sign nonlinearity,” provides the binary outcome indicating whether, at a specific time, the participant is smoking or not. The response of the identified system at a specific time t, $y_{\rm{system}}(t)$, has a nonlinear relation with smoking which is described as

1 $$\left\{ {\begin{array}{*{20}{c}} {{\rm{smoking}}\;(t) = 1\quad \quad {\rm{if}}\,y_{\rm{system}}(t) \ge 1} \\ {{\rm{smoking}}\;(t) = 0\quad \quad {\rm{if}}\,y_{\rm{system}}(t) < 1} \end{array}} \right.$$

In Eq. (1), the use of a threshold equal to 1 is arbitrary. Scaling the threshold will lead to an inverse scaling of the output of the linear system; therefore, since we are dealing with linear systems, it does not change the identification problem or overall results. The same modeling strategy was then deployed on usable data from each participant separately. Next, we simulated how each participant would respond to a specific continuous stress episode lasting 10 min (an arbitrary value to standardize the comparison among participants), to illustrate how these response patterns vary across participants.

**Fig. 4:**
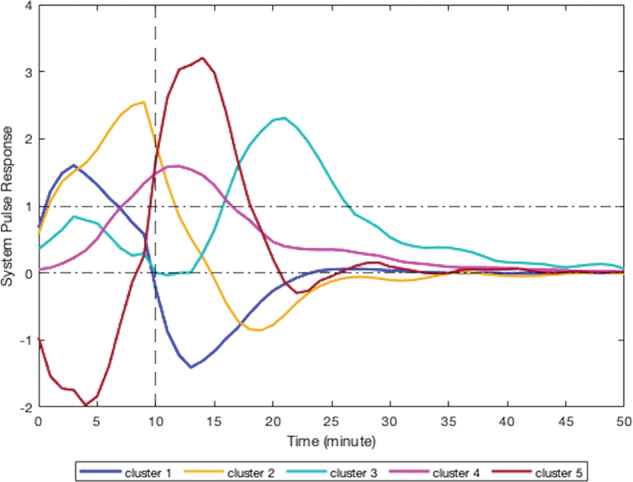
Pulse response of a representative of all clusters. The vertical line at the 10-min mark on the *x*-axis reflects the time when the stress probability returned to zero.

**Fig. 5:**
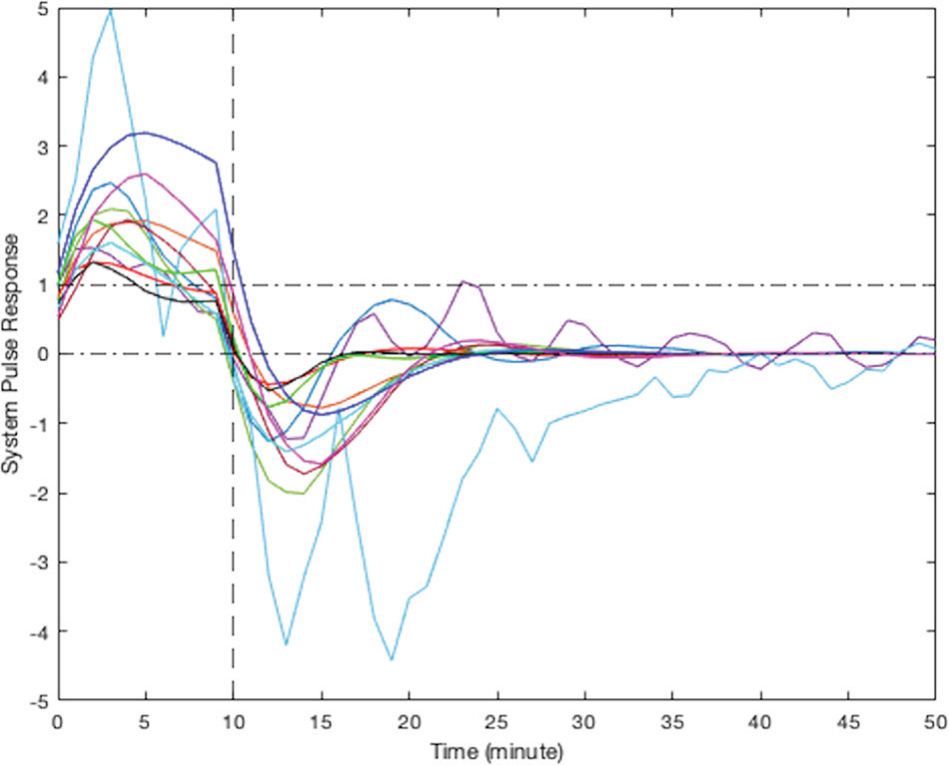
Pulse response for participants in cluster 1, instant increase followed by a sharp decrease in smoking odds. The vertical line at the 10-min mark on the *x*-axis reflects the time when the stress probability returned to zero.

**Fig. 6:**
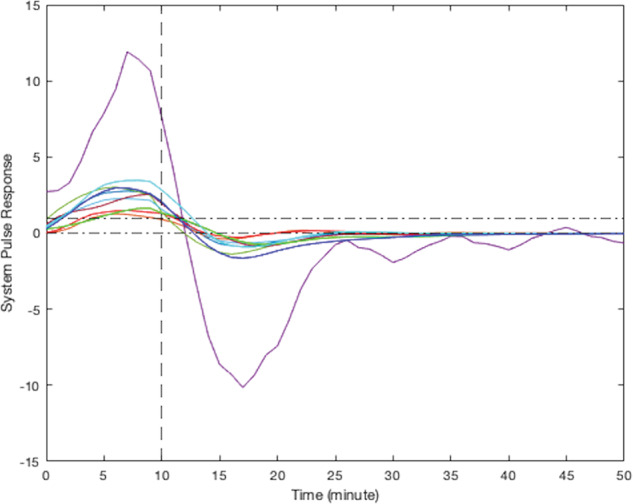
Pulse response for participants in cluster 2, a delayed increase followed by a sharp decrease in smoking odds. The vertical line at the 10-min mark on the *x*-axis reflects the time when the stress probability returned to zero.

**Fig. 7:**
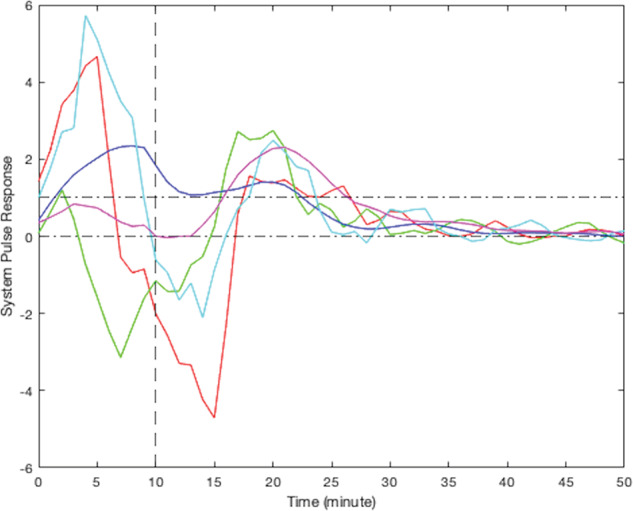
Pulse response for participants in cluster 3, two rounds of increases in smoking odds. The vertical line at the 10-min mark on the *x*-axis reflects the time when the stress probability returned to zero.

**Fig. 8:**
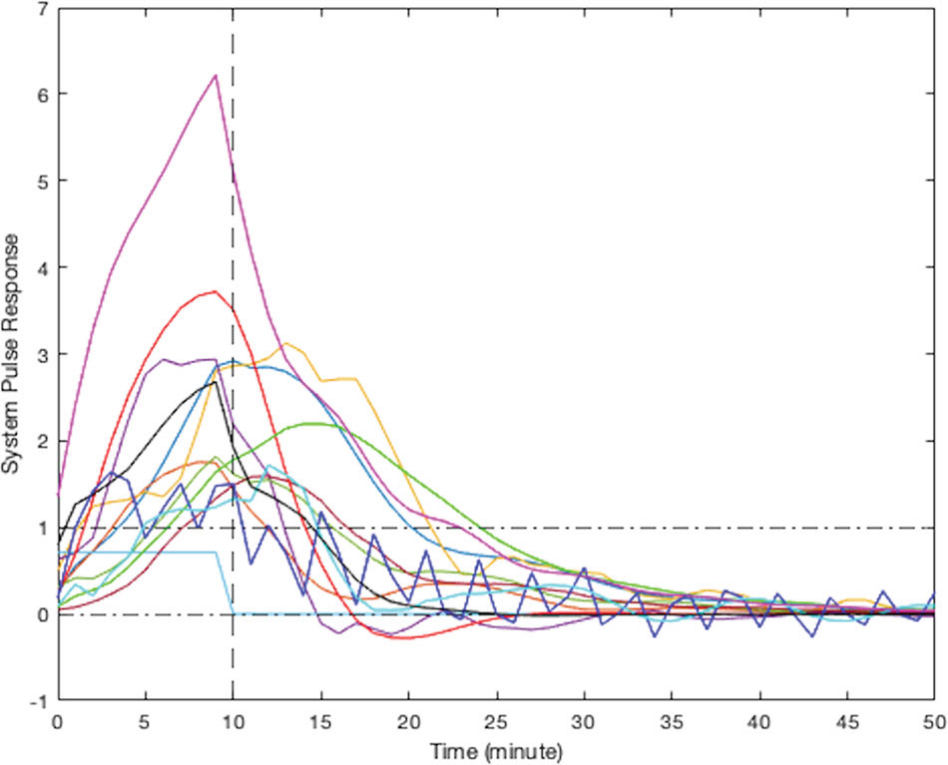
Pulse response for participants in cluster 4, just one delayed increase in smoking odds in response to stress. The vertical line at the 10-min mark on the *x*-axis reflects the time when the stress probability returned to zero.

**Fig. 9:**
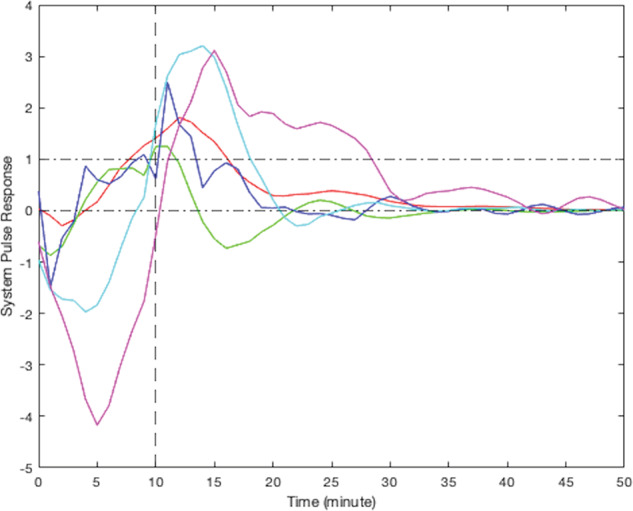
Pulse response for participants in cluster 5, instant decrease in the odds of smoking followed by a sharp delayed increase. The vertical line at the 10-min mark on the *x*-axis reflects the time when the stress probability returned to zero.

According to their respective simulated pulse responses (responses to 10 min of continuous stress), results from the modeling of smoking behavior due to stress for all participants can be divided into five clusters. Due to the limited sample size of this study, visual clustering (i.e., qualitative grouping by the investigators based on the shape, peak delay, and decay to zero for each curve) was applied based on the shape of observed participants responses. Figure 4 presents the simulated responses of a representative participant from each cluster to a 10-min stress episode and Figs. 5–9 represent the pulse responses of individual participants in each cluster to the stress episode, respectively. Cluster 1 was characterized by instantaneous or relatively rapid responses to stress as indicated by an immediate increase in the odds of smoking followed by a sharp decrease in odds of smoking. Cluster 2 was characterized by a delayed response to stress, approximately 7–12 min after the start of the stress episode. Participants in this cluster had an increase and subsequently a decrease in their odds of smoking. Cluster 3 was characterized by an instant increase followed by a second, delayed increase in the odds of smoking due to stress. Cluster 4 was characterized by a single delayed increase in response to stress without a subsequent sharp decrease in the odds of smoking. Finally, cluster 5 was characterized by an instant decrease in the odds of smoking followed by a sharp delayed increase in its odds. It should be noted that the term “delay” implies the time shift in responding. Table 2 summarizes the number of participants belonging to each cluster. It should be noted that the higher magnitudes of the pulse response do not necessarily imply a higher likelihood of smoking. In fact, the magnitude of the pulse response represents the estimated changes of the propensity for smoking when stress happens, as a function of time. If the magnitude is positive, it represents an increase in the likelihood of smoking and if it is negative, it shows a decrease in the likelihood of smoking.

**Table 2:** Number of participants and the percentage for each detected cluster.

Cluster	Number of Participants	Percentage
Cluster 1: immediate increase followed by a decrease in odds of smoking	12	26.7%
Cluster 2: a delayed response to stress, an increase followed by a decrease in odds of smoking	10	22.2%
Cluster 3: two rounds of increase; an instant increase followed by a delayed increase in odds of smoking	5	11.1%
Cluster 4: just a delayed increase in odds of smoking	13	28.9%
Cluster 5: an instant decrease followed by a sharp delayed increase in odds of smoking	5	11.1%

As shown by the results, all pulse responses to a continuous stress episode demonstrated an increase in the odds of smoking either immediately or with some delay. For the majority of participants (*n* = 25), the increase was followed by a sharp decrease in the tendency to smoke, down to negative probability. This can be interpreted as smoking due to stress leading to reducing the probability of further smoking in the near future for these participants. However, some participants (*n* = 12) did not follow the same trend, i.e., the increase in the odds of smoking did not proceed with a decrease in the smoking likelihood in the near future. For a few individuals (*n* = 3), the early increase in the odds of smoking was followed by a second increase in its odds, which showed that smoking in response to stress elevated the odds for further smoking for some people. For some participants (*n* = 5), the behavior was quite different. They represented a negative response to stress at the beginning which can be interpreted as a decrease in the chance of smoking at the first moments of being under stress. The trend is followed by a sharp delayed increase in the odds of smoking. This may indicate the participants’ effort to prevent smoking at first, but eventually stress causes smoking to happen.

## Discussion

This work makes two key contributions toward understanding the relationship between stress and smoking. First, it provides proof of concept for using system identification tools from control systems engineering to identify person-specific dynamic models of stress-smoking responses for participants. In doing so, it introduced a method for combining a linear system with a sign nonlinearity to enable modeling of a continuous predictor (stress) and a binary outcome (smoking). Second, the results of simulations based on person-specific models demonstrated heterogeneity in smoking responses to stress. For about half of the participants (49%), following an initial stress-induced increase in the likelihood of smoking, we expect to see an eventual reduction in the odds of smoking.

This paper demonstrated how dynamical systems modeling techniques can be applied to characterize the dynamics of stress and smoking. This modeling approach accounted for the influence of both recent stress and smoking to predict the odds of smoking in response to future stress. Prior efforts to link stress and smoking have been based on infrequent stress and smoking assessments for each smoking assessment^10,16,42,43^. As sensing technology and digital markers improve, intensive longitudinal data will become more readily available to and important for researchers. Dynamical systems models can incorporate that information to predict the dynamics of smoking systems as a function of stress and other predictor variables.

Another tool for modeling longitudinal data is the time-varying effect model (TVEM)^44–50^. This model has two noteworthy limitations. First, the TVEM cannot determine complex underlying within-time associations of variables^51^, so it is not useful for investigating the long-term effect of predictors on an outcome. TVEM is limited to contemporaneous relations in a single model without considering the effect of delay in that association. That is, TVEM is not capable of exploring the memory effect of stress on smoking. Tools such as dynamical system modeling techniques from control systems engineering can fill this gap when investigators seek to understand the delayed effect of stress on smoking. Second, the TVEM represents bivariate associations as a function of time, but requires an assumption that those time-varying associations are equivalent across people. The dynamical modeling technique applied in this paper was used to generate person-specific models, and the results obtained would not support assumptions of equivalence across participants.

It is worth noting that the dynamical modeling approach applied here was a powerful technique considering the relatively limited amount of time-series data, 3 days in this dataset. Artificial intelligence techniques, including machine learning^52,53^ and deep learning methods^54^, or statistical techniques such as Bayesian approaches and stochastic approximation expectation-maximization algorithms^55,56^ require a large volume of data. In addition, the majority of these methods only provide the ultimate result not the dynamics of the system, that is, predicting whether the participant smokes or not by the end of the stress episode. With the 3 days of data available in this study, these alternative methods were not possible. Thus, control systems engineering tools strike a balance between capitalizing on intensive longitudinal data to reveal dynamics and being efficient in the volume of data required.

The person-specific models describing stress-smoking systems were used to simulate smoking dynamics following a 10-min stress response. In those simulations, stress consistently increased the subsequent likelihood of smoking, but there was considerable variation in how long it took for the likelihood of smoking to increase. In other words, although some participants did not show an immediate increase in smoking likelihood in response to stress, all participants were observed to increase smoking within some minutes of the stress episode. These findings are consistent with the hypothesis that stress is a key risk factor for smoking and some people use smoking to regulate stress^6,10,16,42,43^. Although stress consistently increased the likelihood of smoking, participants varied in the delay between the onset of stress and observed smoking behavior. The latency of stress effects on smoking is an important contribution about which theories have been silent. For some individuals, stress responses have a relatively rapid effect on smoking behavior that may preclude a just-in-time intervention triggered by stress detection; for others, stress responses have a more delayed effect that represents a period of vulnerability when an intervention might be beneficial. Data from this study do not indicate that participants learned to self-administer nicotine to regulate stress responses. Nevertheless, the approach demonstrated here can be extended to multiple time scales to test self-medication hypotheses. Models on faster time scales can characterize stress effects on smoking, and models on slower time scales can characterize how stress responses to smoking affect future stress-smoking dynamics (i.e., learning to self-administer nicotine to regulate stress)^57,58^. Future research could also use *n*-of-1 or single-case research designs to compare system dynamics (e.g., latency of smoking following a stressor) following the introduction and removal of stress-management interventions^59,60^. These approaches would capitalize on the idiographic nature of the systems but allow quasi-experimental comparisons of clinically meaningful outcomes before and after the introduction of an intervention. For example, *n*-of-1 trials could be applied to determine system properties that are amenable or hostile to just-in-time stress-management prompts (e.g., how long of a delay between stress and smoking is required to intervene?).

The results also can be used to inform the selection and timing of just-in-time intervention approaches. Participants in clusters 1 and 2 responded to stress so quickly that they may not have sufficient time to implement a stress-management intervention and regulate themselves before a smoking response is initiated. For them, it may be more effective to implement an intervention aimed at developing stress-management skills that could be employed without a prompt or to tailor just-in-time interventions on other advanced triggers for smoking, such as location or social context. In contrast, participants in clusters 4 and 5 exhibited somewhat slower responses to stress that peaked toward the end rather than the beginning of the stress episode. That delay may provide enough time to detect stress, deliver a prompt to engage in a stress-management exercise (perhaps mediated by an app on the user’s device), improve their regulation, and reduce smoking risk. Participants in cluster 3 had a more complex response. Their risk of smoking increased quickly after the beginning of a stress episode, but it then subsided before increasing again long after the stress episode ended. They may require a more complex intervention with multiple tailoring variables. For example, one tailoring variable could be used to identify moments of anticipated vulnerability and trigger intervention options that reduce the risk of smoking expected shortly after the onset of (anticipated) stressful experiences (e.g., upon arriving at work, sending the user a reminder to seek out supportive coworkers). A second tailoring variable could be used to trigger an intervention option that reduces the risk of smoking after a detected stressful experience has ended (e.g., after detecting a stress response from an upsetting interaction with coworkers, prompting the user to engage in mindfulness practice). This approach of system identification provides a complement to micro-randomized trials for determining the optimal level of individual tailoring variables for triggering different intervention options^61^. These results also suggest that just-in-time stress-management interventions for smoking cessation may have considerable heterogeneity of treatment effects if response systems are not identified to determine the appropriate tailoring variables for a particular participant.

Participants in this study were smokers from a large metropolitan area in a Western country. The stress-smoking systems identified here may not generalize to other contexts or populations. In addition, the smokers in this study were preparing for a quit attempt. Stress-smoking system dynamics may differ for smokers who are not intending to quit or during a quit attempt when withdrawal symptoms present an additional demand. The small sample size was suitable for establishing proof of concept for modeling person-specific changes in smoking responses to stress that can be used to develop person-specific decision rules for just-in-time stress-management interventions; however, the sample size was insufficient to permit empirical clustering of participants based on their responses to stress. Future studies with larger samples can apply clustering algorithms and machine learning techniques like Shapelets to establish a robust typology of stress-smoking systems^62,63^. The model tested was an open loop in nature, meaning it focused on how stress influenced subsequent smoking odds, but did not include feedback from smoking to subsequent stress. A unified closed-loop model was not estimated because we had limited information about other causal influences on stress, but could be a focus for future work with other datasets. In addition, the size of the available dataset limited our ability to apply the validation technique here; however, the validation process is explained in the “Methods” section for use with larger datasets in future studies.

The volume of missing data in the datasets was another limitation of this study. Some missing data were anticipated because the cStress marker cannot be estimated when the participant was physically active. Other missing data was caused by human factors or device issues, such as device non-wear or poor attachment of sensors to the skin. One consequence of the missing data was that each participant’s data were a collection of shorter chunks of data instead of a single long time series. This problem was partially mitigated in the modeling by considering different intrinsic responses for each chunk according to its own past; however, conclusions cannot be drawn about system dynamics between the available chunks, where data were not available. Related to this limitation, the cStress and puffMarker were both informed by a shared sensor (i.e., respiration-inductive plethysmograph) and some shared features of data from that sensor (e.g., inhalation duration). The dynamic nature of the models limited collinearity between the predictor and outcome. Adding additional sensors to provide wholly independent data streams would add a burden for participants, but is worth exploring in future research. Neither marker in this study was perfectly accurate in prior work so caution is warranted when interpreting the results.

A final modeling-related limitation was that we assumed the system is stationary. The dataset did not include information on situational factors that might alter dynamics (e.g., home versus work, weekday versus weekend). Given the relatively short period of 3 days of pre-quit data, we believe it was reasonable to assume that participant behavior would not change dramatically during this period. Accessing and analyzing more data including situational and contextual factors and exploring how they impact smoking should be a priority for future studies.

This study provided proof of concept for using system identification tools from control systems engineering to identify relations between stress and smoking for regular smokers over time. The study made both substantive and methodological contributions to the addiction literature. From a substantive perspective, the identified person-specific dynamical models revealed a direct correlation between stress and subsequent increases in smoking risk. This general pattern was uniform, but the latency between stress and smoking events varied from instantaneous to delayed. In addition, for about half of the participants, smoking in response to stress was followed by a marked reduction in the odds of smoking in the near future. From a methodological perspective, the system identification method yielded person-specific dynamical models that might have a relatively consistent pattern for a given individual, but different patterns for different people. This method provides a new approach for both (a) characterizing the dynamics of smoking risk at different stages (e.g., prior to a quit attempt) and (b) developing person-specific decision rules for just-in-time interventions that support smoking cessation.

## Methods

### Overview of design

This study is a secondary analysis of a subset of data from 45 participants with sufficient sensor data from the Sense2Stop trial conducted through the MD2K Center of Excellence^64^. The Sense2Stop trial involved 15 days of data collection: 3 days prior to a quit attempt, a quit day, and 11 post-quit days. This study used data from the three pre-quit days to develop dynamic models of stress and smoking. All procedures were approved by the IRB at Northwestern University (#STU00201566).

### Participants

Fliers posted in the community were used to recruit adult smokers interested in quitting smoking for the Sense2Stop trial. Prospective participants (*N* = 371) were screened for eligibility and 75 eligible participants enrolled. Participants were between 18 and 65 years, lived in the Chicago area, smoked more than one cigarette per day on average in the past year, were willing to abstain from non-cigarette tobacco products for the course of the study, willing to try to quit smoking for at least 48 h (the parent trial focused on smoking cessation, but the present study was limited to data from the pre-quit period), not taking any medications for smoking cessation currently or planned in the next 30 days, not pregnant or trying to get pregnant, and willing to provide an emergency contact, social security number, and address for study payment. Of the 75 total participants, data preprocessing revealed that 45 participants had sufficient data to estimate person-specific dynamical models of stress-smoking systems (details below).

### Measures

Stress was operationalized as the minute-level probability of stress determined using the cStress algorithm^38^. The cStress marker uses data from a 2-lead electrocardiograph and a respiration-inductive plethysmograph collected by chest-worn AutoSense sensors to estimate the probability of stress at each minute. Specific features used to construct minute-level stress probabilities from those sensors included the 80th percentile and mean of the interbeat interval, and mean and median of the ratio between inspiration and expiration duration. Stress probabilities were calculated every minute, except when accelerometers in the chestband recorded physical activity (due to confounding factors from the physiological responses to activity) or when data were unavailable due to poor sensor attachments or other noise. This cStress model previously demonstrated a true-positive rate of 88.6% and false-positive rate of 4.65% on a test dataset from the lab. The cStress model achieved a median accuracy of 90% and 72% with self-reports from the lab and the field, respectively^38^.

Smoking behavior was operationalized every minute as a binary score from the puffMarker algorithm^39^. This algorithm uses two sources of data: breathing dynamics collected from the respiration-inductive plethysmograph via the AutoSense chestband, and arm movements gathered from inertial sensors (MotionSense) worn on each wrist. Puffs were identified from patterns of hand-to-mouth gestures and respiration cycles. Features extracted consisted of inhalation and exhalation duration, respiration duration, minimum and maximum of the rate of change signal (first derivation of the respiration signal), and mean, median, standard deviation, and quartile deviation of magnitude of gyroscope, pitch, and roll. Excluding isolated puffs, smoking events were marked if a minimum of four smoking puffs was detected. The puffMarker algorithm achieved a true-positive rate of 96.9% and a false-positive rate of 1.1% on the training dataset^39^.

### Procedures

Participants provided written informed consent and were provided with four devices. They were asked to wear two MotionSense wristbands and one AutoSense chestband for up to 16 h/day, and to carry an Android smartphone^65,66^ for the same period. The Autosense chestband supports a wireless sensor suite that records continuous physiological measurements consisting of an electrocardiogram (64 Hz), respiratory inductive plethysmograph (21.3 Hz), and 3-axis accelerometer (10.7 Hz)^65^. Each MotionSense wristband contained a 3-axis accelerometer (16 Hz) and a 3-axis gyroscope (32 Hz) to record arm motion^39,66^. The open-source mCerebrum software has been used to support continuous sensor data transfer from the chestband and wristbands to the smartphone^67^. Participants were compensated if they completed lab visits, responded to mCerebrum-triggered ecological momentary assessments (with bonus for wearing the sensors), and received an overall bonus incentive for wearing the devices more than 70% of the requested wear time during the study period (i.e., 11.2 h/day); however, due to either battery issues or incorrect device wear, the overall wear time was less than that for many participants. Full procedures for the Sense2Stop trial are available from Battalio et al.^37^.

### Analyses

#### Data quality screening

Data were recorded from 75 participants, but participants were excluded if they were part of the pilot test (*n* = 5) or had excessive missing sensor data due to physical activity, improper attachment, non-wear, sensor failures, or insufficient data (i.e., <5 min of continuous data). After accounting for these problems (*n* = 25), the analytic dataset comprised 45 participants (60%).

#### Data processing

Data preprocessing consisted of five general stages: correcting sample times, handling small intervals of missing data via linear interpolation, determining ending time of smoking events, dividing data into continuous pieces called data chunks, and excluding data chunks with less information is done.

First, the timing for each stress probability and the smoking event was provided in timestamp (Unix) format. Therefore, the primary step in processing the data was to convert timestamps to datetime. Datetime represents the time and date information of the events more intuitively, making the results and their display more easily interpretable. The sampling rate in this study was approximately 1 min; however, the timing of some samples violated this criterion, such as when two probabilities were provided for a minute (e.g., with a time difference of 59 s). To address this matter, the approach taken is to round time to the nearest minute. Therefore, in this context, a *sample* means 1 min of information, and a *chunk* is a continuous series of samples.

Second, different durations of missing data occurred in the stress probability measure, ranging from 1 to about 120 min. If intervals of missing data were brief (≤2 min), a linear interpolation technique was used. The reason for this choice is that, given our dataset, two interpolated samples can still provide good consistency as well as maintaining the accuracy of the provided data. Also, these short intervals of missing data would mostly occur as the result of mis-attached sensors on the body rather than any kind of confounding activities that can interfere with stress probability estimation. If intervals were large (>2 min), there was not enough information to make any prediction/estimation of missing stress probabilities at those time intervals.

Third, the puffMarker algorithm identified the start timing of each identified smoking event and the timing of all detected smoking puffs, but did not identify when smoking events ended. A minimum of four puffs in a 5-min time interval was required to qualify as a smoking event. To determine the duration of the smoking events, a sliding 5-min window was used beginning at the start of the episode. As long as the criterion of four puffs inside the considered window was met, the smoking event was extended. Smoking event durations for participants ranged from 2 to 10 min.

Finally, given the fragmented nature of the stress probability dataset and the fact that no information was available to understand relations between stress and smoking during time gaps, we considered each continuous interval of data samples as one “chunk” of data. Some data chunks in our dataset consisted of a very small number of samples, as low as just one sample, which makes it hard for the system to understand the behavior and identify the meaningful relation between stress and smoking. To extract relevant information, chunks with less than five minutes of information were excluded. Removing these small data chunks from the analytic dataset had minimal impact on the number of minutes that could be modeled (on average, 68 min/participant).

#### System identification methods

Solving the problem of identifying parsimonious models (i.e., here estimating the model coefficients describing the dynamics of stress and smoking) from experimental data has led to much recent work. The nonconvex nature of this problem has directed the proceeding approaches^68,69^ to consider relaxations such as Group Lasso or nuclear norm minimization. Although these approaches perform well, they might not be computationally efficient. This difficulty has led to a new approach called atomic norm minimization^70^. This technique represents the response of a linear time-invariant system as a linear combination of suitably chosen objects or atoms^71–73^. Atomic norm minimization results in the efficient identification of sparse models from experimental data. In this paper, we developed the identification algorithm using the atomic norm method. To identify the system (again, to estimate the model coefficients describing the dynamics of stress and smoking), we rely on the idea that the response of a linear time-invariant system can be represented as a linear combination of atoms.

Discrete-time linear system modeling. Note that since we are applying the tools from control systems engineering, in the following, we will use the common terms using in this field. Therefore, from now on “input” refers to the predictor, “output” refers to the outcome, and “*y*_Natural_” represents the intrinsic response.

The response of the linear system, $y\left( k \right)$, is the sum of the response of the system to the input (i.e., stress probability) and the natural response. It can be represented as follows:

2 $$\begin{array}{l}y_{\rm{System}} = y_{\rm{Input}} + y_{\rm{Natural}}\\ \,y\left( k \right) = (h \ast u)(k) + y_N(k)\end{array}$$

where $k$ is the time instant, $u$ and $h$ are the input and impulse response of the system, respectively. $h \ast u$ is the response of the system to the input when the system is starting at rest and $y_N$ is the response of the system while input is considered to be zero. The operator “∗” denotes convolution and can be expanded as below

3 $$\left( {h \ast u} \right)\left( k \right) = \mathop {\sum }\limits_{t = 0}^k h\left( t \right)u(k - t).$$

Any impulse response of a linear time-invariant system can be represented as

4 $$h\left( k \right) = \mathop {\sum }\limits_{i = 0}^{N_p} C_i\alpha _ip_i^k$$

where $C_i$’s are the unknown coefficient of system related to input response, $p$’s are “poles” of the system inside the unit circle, $N_p$ is the number of poles, and $\alpha _p$ is a scaling factor. The scaling term can be represented as

5 $$\alpha _p = \frac{{1 - \left| p \right|^2}}{{1 - \left| p \right|^{N_l + 2}}}$$

where $N_l$ is the length of the measurements vector and $| \cdot |$ returns the absolute value. The size of vector $\alpha _p$ is the same as vector $p$, consisting of poles of the system. Details of this choice of scaling factor is provided in ref. ^72^.

**Remark** The number of poles used here is equal to the order of the system or the complexity of the system.

The response of the system in Eq. (2) can be rewritten as

6 $$y\left( k \right) = \left( {\left( {\mathop {\sum }\limits_p C_p\alpha _pp^k} \right) \ast u} \right)\left( k \right) + \mathop {\sum }\limits_p C_p^N\alpha _pp^k$$

where $\alpha _pp^k$ are the atoms and $C_p$ and $C_p^N$ are the unknown coefficients of system related to input response and natural response, respectively. A precise description of linear systems concepts is represented in Lathi and Green^41^.

#### Data chunks

Considering the dataset as data chunks results in having different intrinsic behaviors with respect to each of the data chunks. Equation (6) can be rewritten for each data chunk as follows:

7 $$y_j\left( m \right) = \left( {\left( {\mathop {\sum }\limits_p C_p\alpha _pp^m} \right) \ast u_j} \right)\left( m \right) + \mathop {\sum }\limits_p C_{jp}^N\alpha _pp^m$$

where *j* represents data chunk *j*, $m$ is the time instant in data chunk *j* and $y_j$, $u_j$, and $C_{jp}^N$ are the system response, input, and coefficients related to natural response for data chunk *j*.

It should be noted that as our dataset is the integration of data chunks with various lengths, the value $N_l$ in Eq. (5) is considered to be the average length of all data chunks for each participant.

We should mention that the value of $y\left( k \right)$ is not provided, but the output of the system which is smoking is available and has a nonlinear relation with $y\left( k \right)$, which is described as

8 $$\left\{ {\begin{array}{*{20}{c}} {y\left( k \right)\, \ge \,1\quad \quad {\rm{if}}\,{\rm{and}}\,{\rm{only}}\,{\rm{if}}\,{\rm{smoking}}\;(k) = 1} \\ {y\left( k \right)\, <\, 1\quad \quad {\rm{if}}\,{\rm{and}}\,{\rm{only}}\,{\rm{if}}\,{\rm{smoking}}\;(k) = 0} \end{array}} \right.$$

Therefore, we use input stress probability and output smoking to identify the system.

#### Sparsity

The objective here is to identify a sparse model in order to minimize the complexity. In other words, we seek to minimize the number of nonzero coefficients in the system, *C*_*p*_’s, and correspondingly maximize the sparsity of the identified system. The literature on sparsity can be found in ref. ^70^.

**Fig. 10:**
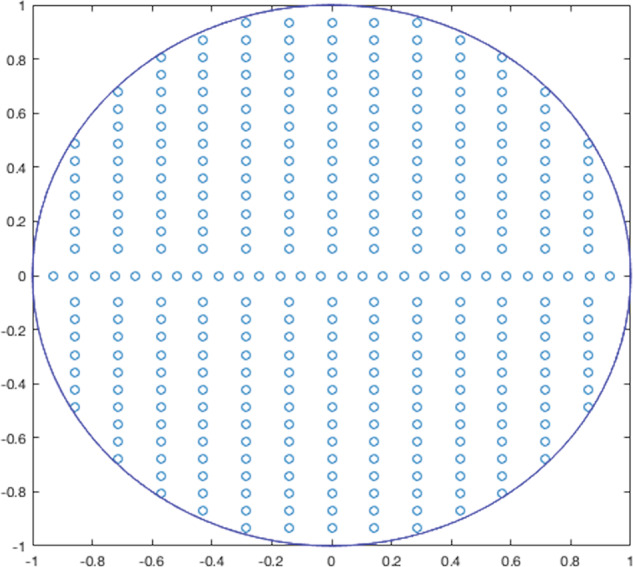
Discretization of a unit circle provides an example of gridding the unit circle used in the software.

For the poles, the unit circle has been gridded uniformly and random poles were picked to check as candidates for the system. Figure 10 represents an example of a gridded unit circle.

#### System identification formulation

The identification problem is formulated as

9 $$\begin{array}{l}\mathop {{\min }}\nolimits_t \,\mid\mid t\mid\mid_0\\ \,{{{\mathrm{subject}}}}\,{{{\mathrm{to}}}}\\ \,\left| {C_p} \right| \le t_p\,\,{\rm{for}}\,{\rm{all}}\,p \in {\rm{Grid}}\\ \,\left| {C_p^{N,l}} \right| \le t_p\,l = 1,2, \ldots ,N_{\rm{ch}}\,\,{\rm{for}}\,{\rm{all}}\,p \in {\rm{Grid}}\\ \,C_p = C_{p^ \ast }^ \ast \\ \,C_p^N = C_{p^ \ast }^{N^ \ast }\,{{{\mathrm{for}}}}\,{{{\mathrm{all}}}}\,{{{\mathrm{data}}}}\,{{{\mathrm{chunks}}}}\\ \,\left\{ {\begin{array}{*{20}{c}} {y\left( k \right) - 1 \ge \varepsilon \,\, {\rm{if}}\,s\left( k \right) = 1} \\ {y\left( k \right) - 1 \, < \, \varepsilon \,\,{\rm{if}}\,s\left( k \right) = 0} \end{array}} \right.\\ \,y\left( k \right) = \left( {\left( {\mathop {\sum }\limits_p C_p\alpha _pp^k} \right) \ast u} \right)\left( k \right) + \mathop {\sum }\limits_p C_p^N\alpha _pp^k\end{array}$$

where $N_{\rm{ch}}$ is the number of data chunks and $\mid\mid t\mid\mid_0$ is $l_0$norm, which is the number of nonzero elements in vector $t$. Minimizing $\mid\mid t\mid\mid_0$ subject to constraints is a computationally complex optimization problem. Therefore, the system identification problem can be solved using $l_1$norm relaxation as follows:

10 $$\begin{array}{l}\mathop {{\min }}\nolimits_t \,\mid\mid t\mid\mid_1\\ {{{\mathrm{subject}}}}\,{{{\mathrm{to}}}}\\ \left| {C_p} \right| \le t_p\,\,{\rm{for}}\,{\rm{all}}\,p \in {\rm{Grid}}\\ \left| {C_p^{N,l}} \right| \le t_p\,l = 1,2, \ldots ,N_{\rm{ch}}\,\,{\rm{for}}\,{\rm{all}}\,p \in {\rm{Grid}}\\ C_p = C_{p^ \ast }^ \ast \\ C_p^N = C_{p^ \ast }^{N^ \ast }\,{{{\mathrm{for}}}}\,{{{\mathrm{all}}}}\,{{{\mathrm{data}}}}\,{{{\mathrm{chunks}}}}\\ \left\{ {\begin{array}{*{20}{c}} {y\left( k \right) - 1 \ge \varepsilon \,\,{\rm{if}}\,s\left( k \right) = 1} \\ {y\left( k \right) - 1 \, < \, \varepsilon \,\,{\rm{if}}\,s\left( k \right) = 0} \end{array}} \right.\\ y\left( k \right) = \left( {\left( {\mathop {\sum }\limits_p C_p\alpha _pp^k} \right) \ast u} \right)\left( k \right) + \mathop {\sum }\limits_p C_p^N\alpha _pp^k\end{array}$$

where $\mid\mid t\mid\mid_1$ is the sum of the absolute values of elements in vector $t$, $s$ is smoking, $p^ \ast$ is the complex conjugate of $p$, and $C_{p^ \ast }^ \ast$ and $C_{p^ \ast }^{N^ \ast }$ are the complex conjugate of $C_p$ and $C_p^N$. $\varepsilon$ is the small margin to make the optimization problem well posed and is considered $10^{ - 5}$ in the implementation. The preprocessing and modeling is done in MATLAB environment and CVX, MATLAB software for disciplined convex programming, is used as the convex optimization toolbox^74^.

#### Validation

In order to validate the models identified in this study, larger datasets are needed for each individual. If such data were available, the following procedure could be applied to validate the developed model for each participant. First, the preprocessed data should be divided into modeling and validation datasets. One approach can be to divide the data with a 2:1 ratio between the modeling and validation groups. Considering the variations in the number of data points in each chunk, the 2:1 ratio might be adjusted by a few data points, if data points are not the same in each chunk. This way, there is no need to divide the middle chunk and the whole chunk will be in one of the categories. Due to the different lengths of data chunks, the number of chunks to be distributed between the two groups may be uneven.

As the second step, for the modeling data, the optimization problem (10) should be solved to identify the dynamic models for the individuals. Solving this problem identifies the coefficients of the system for the input and natural responses and provides the impulse response and poles of the system that can be used for the validation step. One of the common methods to validate a model is to assess how closely the model outcomes match the observations by measuring the size of prediction error between the two. Therefore, having the impulse response and poles of the system from the second step, for validation, an optimization problem should be solved to identify the coefficients of the natural response by minimizing the size of the error. Similar to the approach used in solving the system identification problem for the modeling data, this problem can also be solved using $l_1$norm relaxation

11 $$\begin{array}{l}\mathop {{\min }}\nolimits_{C_p^N} \,\mid\mid\mid\mid_1\\ {{{\mathrm{subject}}}}\,{{{\mathrm{to}}}}\\ C_p^N = C_{p^ \ast }^{N^ \ast }\,{{{\mathrm{for}}}}\,{{{\mathrm{all}}}}\,{{{\mathrm{data}}}}\,{{{\mathrm{chunks}}}}\\ y\left( k \right) = \left( {\left( {\mathop {\sum }\limits_p C_p\alpha _pp^k} \right) \ast u} \right)\left( k \right) + \mathop {\sum }\limits_p C_p^N\alpha _pp^k\\ \left\{ {\begin{array}{*{20}{c}} {y\left( k \right) + \delta (k) \ge 1 + \varepsilon \,\,{\rm{if}}\,s\left( k \right) = 1} \\ {y\left( k \right) + \delta (k) < 1 - \varepsilon \,\,{\rm{if}}\,s\left( k \right) = 0} \end{array}} \right.\end{array}$$

where $C_p^N$ is the unknown coefficients, $\delta (k)$ is the error between the overall model response and the output at time $k$, and $\,\mid\mid\mid\mid_1$ is the sum of the absolute values of $\mid\mid\mid\mid_1$. Solving this optimization problem, if $\delta (k)_1$ is below a threshold (which would vary depending on the number of samples in the dataset), the model and the results are valid.

### Reporting summary

Further information on research design is available in the Nature Research Reporting Summary linked to this article.

## Supplementary information

Supplementary Information

Reporting Summary

## Data Availability

The datasets generated during and/or analyzed during the current study are available from the corresponding author on reasonable request.

## Code availability

Custom code used to generate models reported in this paper are available from https://github.com/Lagoa-s-lab/DOME.
