# Supplementary material for: Dynamic models of stress-smoking responses based on high-frequency sensor data
Source: NPJ Digit Med. 2021 Nov 23;4:162. doi: 10.1038/s41746-021-00532-2 (PMC8611062; doi:10.1038/s41746-021-00532-2)
Supplement: Supplementary file 1 — Supplementary Information [file 41746_2021_532_MOESM1_ESM.pdf]

Supplementary Table 1. Data information for all the participants.

Participant ID	Original data samples (in minutes)	Data samples after interpolation (in minutes)	Number of original data chunks	Number of data chunks after discarding chunks with size < 5	Data samples after discarding data with chunk size < 5 (in minutes)	Cluster
201	529	632	30	20	607	1
202	513	642	63	38	591	1
203	650	775	46	22	736	3
204	434	554	65	30	473	2
205	706	878	81	40	792	1
208	894	1169	121	64	1057	3
209	538	625	38	26	601	1
210	79	90	3	3	90	4
211	953	1182	85	51	1114	1
212	911	1076	89	45	989	1
213	782	913	63	33	860	2
214	768	976	113	50	851	2
215	1176	1380	51	31	1346	1
216	706	889	62	40	846	5
217	852	1043	90	48	957	4
219	766	933	55	37	898	2
220	553	673	66	33	598	5
222	625	770	89	47	699	4
226	1014	1253	101	67	1186	3
228	843	1063	90	50	992	2
229	1452	1776	90	60	1716	2
230	1077	1329	112	60	1237	4
231	1132	1402	148	80	1280	4
233	803	992	98	51	909	4
234	392	550	69	39	496	4
235	802	995	75	36	916	5
240	766	960	120	58	842	1
242	318	458	94	37	355	2
244	356	433	36	19	399	4
245	500	620	74	29	538	4
250	1169	1401	85	52	1345	4
251	1129	1336	95	52	1250	2
252	1221	1459	96	51	1365	5
253	1180	1408	78	56	1366	4
255	611	789	99	47	688	1
257	351	420	12	10	412	1
258	194	236	22	14	221	1
259	310	365	31	17	340	2
260	511	629	64	35	582	2
261	918	1103	56	37	1067	3
262	823	1023	111	60	914	1
264	377	529	150	40	335	4
265	1152	1353	88	55	1293	5
268	590	717	97	40	602	3
270	115	157	26	14	143	4

Supplementary Table 2. Statistics of data used in this study.

Data Characteristic	Mean	Standard Deviation	Median	Minimum	Maximum
Original data samples (in minutes)	723.13	323.57	766	79	1452
Data samples after interpolation (in minutes)	887.91	385.48	913	90	1776
Number of original data chunks	76.15	33.12	81	3	150
Number of data chunks after discarding chunks with size < 5	40.53	16.46	40	3	80
Data samples after discarding data with chunk size < 5 (in minutes)	819.86	375.81	846	90	1716
