# Supplementary material for: Dynamic models of stress-smoking responses based on high-frequency sensor data
Source: NPJ Digit Med. 2021 Nov 23;4:162. doi: 10.1038/s41746-021-00532-2 (PMC8611062; doi:10.1038/s41746-021-00532-2)
Supplement: Supplementary file 2 — Reporting Summary [file 41746_2021_532_MOESM2_ESM.pdf]

nature portfolio

Corresponding author(s): David E Conroy

Last updated by author(s): Sep 15, 2021

Reporting Summary

Nature Portfolio wishes to improve the reproducibility of the work that we publish. This form provides structure for consistency and transparency in reporting. For further information on Nature Portfolio policies, see our Editorial Policies and the Editorial Policy Checklist.

Statistics

For all statistical analyses, confirm that the following items are present in the figure legend, table legend, main text, or Methods section.

n/a	Confirmed	
☐	☒	The exact sample size (n) for each experimental group/condition, given as a discrete number and unit of measurement
☐	☒	A statement on whether measurements were taken from distinct samples or whether the same sample was measured repeatedly
☒	☐	The statistical test(s) used AND whether they are one- or two-sided<br>*Only common tests should be described solely by name; describe more complex techniques in the Methods section.*
☒	☐	A description of all covariates tested
☒	☐	A description of any assumptions or corrections, such as tests of normality and adjustment for multiple comparisons
☐	☒	A full description of the statistical parameters including central tendency (e.g. means) or other basic estimates (e.g. regression coefficient) AND variation (e.g. standard deviation) or associated estimates of uncertainty (e.g. confidence intervals)
☒	☐	For null hypothesis testing, the test statistic (e.g. F, t, r) with confidence intervals, effect sizes, degrees of freedom and P value noted<br>*Give P values as exact values whenever suitable.*
☒	☐	For Bayesian analysis, information on the choice of priors and Markov chain Monte Carlo settings
☐	☒	For hierarchical and complex designs, identification of the appropriate level for tests and full reporting of outcomes
☒	☐	Estimates of effect sizes (e.g. Cohen's d, Pearson's r), indicating how they were calculated

Our web collection on statistics for biologists contains articles on many of the points above.

Software and code

Policy information about availability of computer code

Data collection	Open-source code (mCerebrum and Cerebral Cortex) was used for data collection. This code is available from https://github.com/MD2Korg/.
Data analysis	MATLAB software for disciplined convex programming, CVX, was used for data analysis. The code is available from https://github.com/Lagoa-s-lab/DOME.

For manuscripts utilizing custom algorithms or software that are central to the research but not yet described in published literature, software must be made available to editors and reviewers. We strongly encourage code deposition in a community repository (e.g. GitHub). See the Nature Portfolio guidelines for submitting code & software for further information.

Data

Policy information about availability of data

All manuscripts must include a data availability statement. This statement should provide the following information, where applicable:

- Accession codes, unique identifiers, or web links for publicly available datasets
- A description of any restrictions on data availability
- For clinical datasets or third party data, please ensure that the statement adheres to our policy

Data used for these models are available upon reasonable request from the corresponding author.

Field-specific reporting

Please select the one below that is the best fit for your research. If you are not sure, read the appropriate sections before making your selection.

☐ Life sciences ☒ Behavioural & social sciences ☐ Ecological, evolutionary & environmental sciences

For a reference copy of the document with all sections, see nature.com/documents/nr-reporting-summary-flat.pdf

Behavioural & social sciences study design

All studies must disclose on these points even when the disclosure is negative.

Study description	Quantitative
Research sample	Adult smokers interested in smoking (parent study N=75; analytic sample n=45). Mean age = 42, 51% male, 49% female. The sample is not representative of all smokers.
Sampling strategy	Convenience sample. No power analysis was conducted because inferential statistics were not calculated. Models were person-specific and the sample was selected to include participants with sufficient data to estimate person-specific models.
Data collection	Data were collected using the AutoSense chestband (respiration-inductive plethysmograph, electrocardiogram, accelerometer) with MotionSense (accelerometers, gyroscopes) devices on each wrist.
Timing	February 2017 - August 2019
Data exclusions	Data were recorded from 75 participants, but participants were excluded if they were part of the pilot test (n = 5) or had excessive missing sensor data due to physical activity, improper attachment, non-wear, sensor failures, or insufficient data (i.e. less than five minutes of continuous data). After accounting for these problems (n = 25), the analytic dataset comprised 45 participants (60%).
Non-participation	Of the 75 participants who provided consent and enrolled in the study, 10 dropped out (7 did not want to wear the study devices/sensors, 1 did not want to receive coaching, and 2 did not give a reason for dropping otu)
Randomization	N/A

Reporting for specific materials, systems and methods

We require information from authors about some types of materials, experimental systems and methods used in many studies. Here, indicate whether each material, system or method listed is relevant to your study. If you are not sure if a list item applies to your research, read the appropriate section before selecting a response.

Materials & experimental systems

n/a	Involved in the study	
☒	☐	Antibodies
☒	☐	Eukaryotic cell lines
☒	☐	Palaeontology and archaeology
☒	☐	Animals and other organisms
☐	☒	Human research participants
☒	☐	Clinical data
☒	☐	Dual use research of concern

Methods

n/a	Involved in the study	
☒	☐	ChIP-seq
☒	☐	Flow cytometry
☒	☐	MRI-based neuroimaging

Human research participants

Policy information about studies involving human research participants

Population characteristics	See above
Recruitment	Fliers posted in the community were used to recruit adult smokers interested in quitting smoking.
Ethics oversight	All procedures were approved by the IRB at Northwestern University (#STU00201566).

Note that full information on the approval of the study protocol must also be provided in the manuscript.
